# Postbiotic, anti-inflammatory, and immunomodulatory effects of aqueous microbial lysozyme in broiler chickens

**DOI:** 10.1080/10495398.2024.2309955

**Published:** 2024-02-07

**Authors:** Mustafa Bastamy, Ismail Raheel, Ahmed Elbestawy, Mohamed Diab, Enas Hammad, Lamiaa Elebeedy, Amal M. El-Barbary, Ghadeer M. Albadrani, Mohamed M. Abdel-Daim, Mervat A. Abdel-Latif, Ahmed Orabi

**Affiliations:** a Department of Poultry Diseases, Faculty of Veterinary Medicine, Cairo University, Giza, Egypt; b Department of Bacteriology, Mycology and Immunology, Faculty of Veterinary Medicine, Beni-Suief University, Beni-Suief, Egypt; c Department of Poultry and Fish Diseases, Faculty of Veterinary Medicine, Damanhour University, Elbeheira, Egypt; d Department of Animal Hygiene and Zoonoses, Faculty of Veterinary Medicine, New Valley University, El Kharga, Egypt; e Agricultural Research Center (ARC), Animal Health Research Institute-Mansoura Provincial Lab (AHRI-Mansoura), Dokki, Giza, Egypt; f Faculty of Pharmacy, New Valley University, El Kharga, Egypt; g Poultry Breeding Research Department, Animal Production Research Institute, Agriculture Research Center, Giza, Egypt; h Department of Biology, College of Science, Princess Nourah bint Abdulrahman University, Riyadh, Saudi Arabia; i Department of Pharmaceutical Sciences, Pharmacy Program, Batterjee Medical College, Jeddah, Saudi Arabia; j Pharmacology Department, Faculty of Veterinary Medicine, Suez Canal University, Ismailia, Egypt; k Department of Nutrition and Veterinary Clinical Nutrition, Faculty of Veterinary Medicine, Damanhour University, Damanhour, Egypt; l Department of Microbiology, Faculty of Veterinary Medicine, Cairo University, Giza, Egypt

**Keywords:** Lysozyme, postbiotic, anti-inflammatory, immunomodulation, broiler chickens

## Abstract

Lysozymes, efficient alternative supplements to antibiotics, have several benefits in poultry production. In the present study, 120, one–day–old, Ross 308 broiler chickens of mixed sex, were allocated into 2 equal groups, lysozyme treated group (LTG) and lysozyme free group (LFG), to evaluate the efficacy of lysozyme (Lysonir^®^) usage via both drinking water (thrice) and spray (once). LTG had better (*p* = 0.042) FCR, and higher European production efficiency factor compared to LFG (*p* = 0.042). The intestinal integrity score of LTG was decreased (*p* = 0.242) compared to that of LFG; 0.2 vs. 0.7. Higher (*p* ≤ 0.001) intestinal Lactobacillus counts were detected in chickens of LTG. Decreased (*p* ≤ 0.001) IL-1β and CXCL8 values were reported in LTG. The cellular immune modulation showed higher (*p* ≤ 0.001) opsonic activity (MΦ and phagocytic index) in LTG vs. LFG at 25 and 35 days. Also, higher (*p* ≤ 0.001) local, IgA, and humoral, HI titers, for both Newcastle, and avian influenza H5 viruses were found in LTG compared to LFG. In conclusion, microbial lysozyme could improve feed efficiency, intestinal integrity, *Lactobacillus* counts, anti-inflammatory, and immune responses in broiler chickens.

## HIGHLIGHTS

- Exogenous aqueous and spray microbial lysozyme enhanced growth in commercial broiler chickens
- The postbiotic effects of microbial lysozyme modulated intestinal integrity.
- Anti-inflammatory, as well as local, cellular, and humoral immune response were stimulated by lysozyme supplementation.

## Introduction

For more than 60 years, the poultry industry has utilized antibiotics as growth promoters to boost meat production. Antibiotic-resistant bacteria have been emerged because of this approach, and potentially endangered human health. As a result, non-antibiotic alternatives were urgently needed to sustain poultry health and increase feed benefits ratio.^1–9^ Thus, many attempts were made to improve gut health, modulate microbiota, enhance intestinal integrity, and manipulate the bacterial cecal community in terms of amending chicken growth performance and body weight. The development of dietary supplements including antimicrobial peptides, bacteriocins, probiotics, prebiotics, herbs and exogenous antimicrobial enzymes such as lysozymes have therefore been continuously increased.^10–12^

Lysozymes are common antimicrobial enzymes that were discovered 100 years ago by Alexander Fleming.^13^ These enzymes are widespread in many animal tissues and secretions and the commercial source is obtained from poultry egg white. Different origins of lysozyme exist such as animal, plant, microbial and phage lysozymes.^14^ The primary chemical structure of lysozyme is a single polypeptide chain with 129 amino acids including 4 pairs of cysteine.^15^ The antibacterial actions of lysozyme are exerted through both direct bacterioloytic effect by disrupting 1,4-glycosidic linkage between N-acetylmuramic acid and N-acetylglucosamine of bacterial peptidoglycans in the cell walls of Gram positive and Gram negative bacteria^16^ and indirect stimulation of macrophage phagocytic functions.^17^ Additionally, it supports gut barrier function, thereby improving the growth performance.^18^ Broiler breeder hens’ growth performance, gut microbiota, antioxidant status, and nonspecific immunity were all enhanced by nutritional supplementation with exogenous lysozyme at a dosage of 90 g ton^−1^.^19^ A fusion protein, composed of lysozyme and surfactant protein B, proposed by Akinbi et al.^20^ for prevention or treatment strategy of cystic fibrosis caused by *Pseudomonas aeruginosa* in mice led to 6–30 fold higher bacterial clearance compared to wild-type controls. Several records of significant reduction of pneumonia in human following aerosol administration of lysozyme due to the significant reduction in the pneumonia related parameters, such as the bacterial colony-forming in the whole lung and bronchoalveolar lavage fluid (BALF), and the total BALF leukocytes suggesting the effective mitigation of respiratory disorders.^21^^,^^22^ The effects of spraying lysozyme solutions during the food industry on the microbiological stability and organoleptic characteristics of chicken legs with skin have been described with variable degrees of activity. The findings indicated that during cold storage of the legs, there was a significant suppression of the early aerobic bacterial development alongside a limiting of harmful organoleptic alterations. The initial aerobic bacterial count was reduced by 20 times as a result of the larger dosages of lysozyme solution (48,000 U/mL), which may be useful in increasing the shelf life of portioned poultry meat.^23^

The anti-inflammatory effect mechanisms of lysozymes were proven previously.^19^^,^^24–27^ These studies highlighted the antibacterial, immunomodulatory and systemic anti-inflammatory mechanisms of lysozymes in details. Furthermore, Obmińska-Mrukowicz^28^ studied the immunomodulatory properties of lysozyme dimer in laboratory animals and indicated the pharmacological protection of immunohomeostasis during viral and bacterial infections. Moreover, the researcher mentioned that lysozyme could be applied to enhance the immune response during vaccination and for the compensation of the impaired immune system function due to immunosuppressive factors.

To date, the effect of different routes of lysozyme administration on broiler chickens has not been yet fully investigated. Most of studies often focus on lysozyme administration in feed from birth through market age. However, no studies have also focused on adding lysozyme in drinking water and/or spray that may be appropriate and have the greatest effect on intestinal bacteria populations and broiler performance at exact crucial phases of the broiler growth cycle. Therefore, in this study we aimed to evaluate the postbiotic, anti-inflammatory and immunomodulatory effects of novel aqueous lysozyme (Lysonir®), thrice in drinking water and once as a spray, in commercial broiler chickens.

## Material and methods

### Ethics statement

All experimental procedures were approved by the Animal care committee of Beni-Sueif University, Egypt (approval number: 021-168).

### Experimental design

#### Chickens and dietary treatments

120, One day old, Ross 308 broiler chickens of mixed sex were assigned to 2 separate (equal) groups that were floor reared in 2 separate units each containing 6 replicates of 10 birds. Balanced rations [corn–soybean based that were formulated based on the nutrient requirements for Ross-308 broiler chickens],^29^ were provided as shown in Table 1. Clean drinking water was *ad libitum* offered. The vaccination program for both groups was also as follows: bivalent live infectious bronchitis and Newcastle vaccine, MA5 + Clone 30 (Nobilis® Ma5-Clone30, MSD, Intertvet Int., The Netherlands) at 5 days of age via eye drop (ED), bivalent inactivated avian influenza subtype H5 plus Newcastle vaccine (MEFLUVAC^®^ H5 + ND7, MEVAC, Egypt) at 10 days of age through subcutaneous route with a dose of 0.5 mL/bird, Gumboro intermediate plus, Bursine plus^®^ (Zoetis, USA) at 12 days of age via ED and live Newcastle, laSota^®^ (MSD, Intertvet Int., The Netherlands), at 18 days of age via ED.

**Table 1.** Analysis of the starter and grower diets used in the experiments for the percentage of ingredients and stated composition (%, as-fed basis).

Ingredients %	Starter (0–10 d)	Grower (11–21 d)	Finisher (22–35 d)
Yellow corn	54.78	58.88	63.90
Soybean meal (44%)	33.5	29.4	24
Corn gluten (60%)	5	5	5
Corn oil	2	2.65	3.15
Dicalcium phosphate	1.73	1.6	1.5
Limestone	1.35	1	1
Salt	0.4	0.4	0.4
DL-methionine*	0.15	0.12	0.1
HCl-lysine**	0.35	0.3	0.3
Vitamins and minerals premix ***	0.3	0.3	0.3
Antimycotoxin	0.2	0.2	0.2
Sodium bicarbonate	0.1	0.1	0.1
Choline chloride	0.05	0.05	0.05
Calculated composition			
ME, Kcal/Kg diet	3005	3100	3195
CP%	23	21.5	19.5
Ca%	1	0.87	0.82
Avail. P%	0.47	0.44	0.41
Methionine%	0.56	0.51	0.47
Lysine%	1.44	1.29	1.14
Meth. + Cyst. %	0.93	0.86	0.78
Na%	0.20	0.20	0.20

SBM: soybean meal; ME: metabolizable energy; CP: crude proteins; Av. (P): available phosphorus. *DL—methionine 99% feed grade China; **L—lysine 99% feed grade; ***vitamin and mineral premix (Hero mix) manufactured by Hero pharm and composed (per 3 kg) of vitamin A 12,000,000 IU, vitamin D3 2,500,000 IU, vitamin E 10,000 mg, vitamin K3 2000 mg, vitamin B1 1000 mg, vitamin B2 5000 mg, vitamin B6 1500 mg, vitamin B12 10 mg, niacin 30,000 mg, biotin 50 mg, folic acid 1000 mg, pantothenic acid 10,000 mg, manganese 60,000 mg, zinc 50,000 mg, iron 30,000 mg, copper 4000 mg, iodine 300 mg, selenium 100 mg, and cobalt 100 mg.

The Lysonir^®^ treated group (LTG) was treated by Lysonir ^®^ (20%) [Microbial lysozyme contains 200 g/L of microbial lysozyme extracted from *Acremonium alcalopilum* (the only known cellulolytic saprophytic fungus that thrives in alkaline conditions)], produced by MN Trade Industrial Inc., 10^th^ of Ramadan city, Egypt, under registration code in agriculture ministry 2931], for 8 h daily (from 8 AM to 4 PM) during the first 3 days in drinking water using the recommended dose (0.5 mL/L). The same treatment dose was then repeated at days of 11–13 and 18–20 of age, with a daily treating for 8 h in drinking water. Finally, Lysonir^®^ (coarse spray form; coarse spray with droplet size of 100 microns. Spraying was done over the head of birds with 50 cm height from 8 AM for 10 minutes) at 25–27 of age using the recommended dose of 6.5 mL/200 bird was applied. The second group was reared without addition of Lysonir^®^, free group, (LFG). Birds were exposed to 24 h light throughout the study.

### Postbiotic effect assessments

#### Performance parameters

Average final body weight (AFBW) and feed intake (FI) were weekly determined while feed conversion ratio (FCR) was calculated till the 5^th^ week of age (end of the experiment). The final European production efficiency factor (EPEF) was also estimated using Equation (1):

(1) EPEF=[(average body weight×survival rate)÷(feed conversion rate×experiment′s days)]×100

#### Intestinal length and width

The length of intestine (from duodenum to ileum) and width of the ileum center in 18 sacrificed birds from each group were measured at 35^th^ day of age.^30^ Humanely sacrification through cervical dislocation after the intravenous injection of sodium pentobarbital with a dose of 50 mg/kg was applied.

#### Intestinal integrity score

The small intestine from 18 sacrificed birds of each group at 35^th^ day of age was opened and scored on a scale from zero to four, based on parameters, such as intestinal ballooning; serosal and/or mucosal redness; reduction of intestinal wall thickness; flaccid and fragile intestinal edges within 3 s after gut dissection; abnormal lumen contents (all previous lesions were inspected at the cranial and caudal from Meckel’s diverticulum) and presence of undigested feed particles caudal from ileo-cecal junction. If any of them were present, they received a score of one; otherwise, they receive a score of 0.^31^

#### Intestinal microbiota

Enumeration of lactic acid bacteria (*Lactobacillus* count): one gram of fresh digesta samples from the crop, ileum, and cecum of 18 sacrificed birds in each group at 15 and 35 days was transferred to 9 mL MRS (De Man, Rogosa and Sharpe) broth and serially diluted in 10-fold increments. 0.1 mL from the last three diluted samples were individually plated on MRS agar (Oxoid Ltd., Hampshire, UK), they were then incubated at 37 °C for 48 h (under microaerophilic conditions).^30^

### Inflammatory mediators screening

#### Interleukin-1β (IL-1β) assay

IL-1β concentrations were determined by immunoenzymatical assay using chicken IL-1β enzyme linked immunosorbent assay (ELISA) kits (Novatein Bio, Massachusetts, USA). Eighteen serum samples from each group collected at 7, 15, 25, and 35 days. A wavelength of 450 nm was employed to detect the absorptions and by using software, IL-1β concentrations were determined from the standard curve.

#### RT-qPCR for determination of expression and fold change of CXCL8 and GAPDH genes

Eighteen blood samples from each group, obtained at 7, 15, 25, and 35 days, were examined using RT-qPCR methods to determine changes in the gene expression of CXCL8 in monocyte-derived macrophages. The subsequent primers: CXCL8 F: TAG GAC CAG AGC CAG GAA GA, R: GCT GCA GAA AGC AGG AAA AC, and QuantiTect SybrGreen master mix (Qiagen, Germany) were utilized. Their cycling circumstances were carried out at 95 °C/5 min, 40 cycles of 95 °C/10 s, 60 °C/30 s, and 72 °C/1 min. RT-qPCR results were then analyzed using comparative threshold cycle (CT).^32^

### Immune mediators screening

#### Local and cellular immunity

##### Mucosal IgA

Tracheal IgA were measured in 18 serum samples from each chicken group collected at 7, 15, 25, and 35 days using chicken immunoglobulin A (IgA) ELISA kit (catalog No. E33-112, Bethyl Laboratories Inc., Montgomery, TX, USA) according to the method described by Merino-Guzmán et al.^33^

##### Opsonic activity assay

CytoSelect™ 96-Well Phagocytosis Assay (Red Blood Cell Substrate, catalog No. CBA-220, Cell Biolabs Inc., San Diego, CA, USA) was used for the detection of MΦ count and phagocytic index in 18 serum samples collected from each chicken group at 7, 15, 25 and 35 days of age according to the method described by Yu et al.^34^

### Humoral immunity

#### Hemagglutination inhibition (HI) assay

Eighteen serum samples from each group were collected at 25 and 35 days for the detection of HI antibody titers of highly pathogenic avian influenza (HPAI) H5N1 and Newcastle disease (ND). An inactivated AI-H5N1 antigen (A/chicken/Egypt/18-H/2009) was used as the antigens (Ags) to detect AI-H5 antibodies, where LaSota strain for detection of ND antibodies using 8 HA units of both Ags was also used.^35^

### Statistical analysis

The results were presented as means ± SD using independent *T*-test to determine the significance of differences between LTG and LFG in all mentioned parameters. A probability (*p* values below 0.05 was considered as statistically significant.

## Results

### Postbiotic effect of lysozyme

For the performance parameters as indicated in Table 2, an insignificant decrease (*p* = 0.390) in FI (2520 vs. 2530 g), a significant improved (*p* = 0.042) in FCR (1.36 vs. 1.40), and an insignificant (*p* = 0.288) numerically higher AFBW (1890 vs. 1850 g) were recorded in LTG compared to LFG. The final EPEF was also significantly higher (*p* = 0.040) in LTG compared to LFG (397 vs. 379.5).

**Table 2.** Postbiotic effect of microbial lysozyme on performance.

Item	LTG	LFG	*p*-Value
Average final body weight, g	1890 ± 7.9^a^	1850 ± 12.5^a^	0.288
Feed intake, g	2520 ± 27.9^a^	2530 ± 38.8^a^	0.390
Feed conversion ratio	1.36 ± 0.03^b^	1.40 ± 0.04^a^	0.042
European production efficiency factor	397 ± 5.36^a^	377.5 ± 9.91^b^	0.040

Any two means for a performance parameter bearing different superscript letters in a row are significantly (*p* < 0.05) different from each other.

European production efficiency factor = [(viability % × body weight Kg/age (d) × FCR)] × 100.

The length of intestine (from duodenum to ileum) and width of the ileum center was 175 and 1.45 versus 150 and 1.23 cm in LTG and LFG, respectively. The intestinal integrity score was also significantly decreased (*p* = 0.042) with a value of 0.2 in LTG vs. 0.7 in LFG. Regarding the intestinal *Lactobacillus* count (log_10_ CFU/g), significantly greater (*p* ≤ 0.01) values in the crop, ileum, and cecum were recorded in chickens of LTG at 15 and 35 days, as shown in Table 3.

**Table 3.** Postbiotic effect of microbial lysozyme on the intestinal integrity and intestinal lactobacillus count (log_10_ CFU/g).

Item	LTG	LFG	*p-*Value
Intestinal integrity			
Intestinal length, cm	175 ± 0.2^a^	150 ± 0.3^b^	0.046
Intestinal diameters, cm	1.45 ± 0.02^a^	1.23 ± 0.04^b^	0.001
Intestinal integrity score	0.2 ± 0.004^a^	0.7 ± 0.002^b^	0.042
Intestinal lactobacillus count (log_10_ CFU/g)			
Crop, d 15	4.2 ± 0.25^a^	2.5 ± 0.46^b^	0.001
Crop, d 35	6.85 ± 0.33^a^	4.52 ± 0.45^b^	0.001
Ileum, d 15	5.6 ± 0.24^a^	4.3 ± 0.65^b^	0.019
Ileum, d 35	7.64 ± 0.6^a^	5.65 ± 0.48^b^	0.002
Cecum, d 15	6.2 ± 0.34^a^	5.2 ± 0.43^b^	0.021
Cecum, d 35	8.4 ± 0.28^a^	6.2 ± 0.34^b^	0.008

Any two means for a performance parameter bearing different superscript letters in a row are significantly (*p* < 0.05) different from each other.

### Anti-inflammatory effects

**Figure 1.**
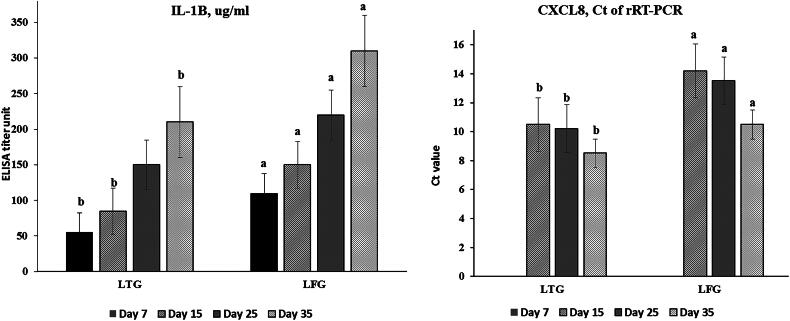
Anti-inflammatory assessments (a: *IL-1β*; b: CXCL8) in both groups. Different letters above the bars indicate statistically significant difference between groups at *p* value below 0.05.

The anti-inflammatory effects of lysozyme were evident through the significant lower (*p* ≤ 0.001) IL-1β and CXCL8 (pro-inflammation indicators) values in LTG rather than LFG which were reported throughout the experiment, (Fig. 1a,b), e.g., at 35 days, results were 210 vs. 310 and 8.5 vs. 10.5 for IL-1β and CXCL8 in LTG and LFG, respectively.

### Cellular and local immunity

**Figure 2.**
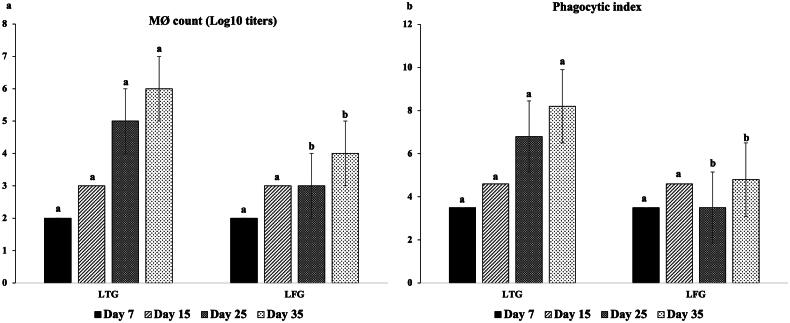
Cellular immune modulation (a: MΦ count; b: phagocytic index) in both groups. Different letters above the bars indicate statistically significant difference between groups at *p* value below 0.05.

**Figure 3.**
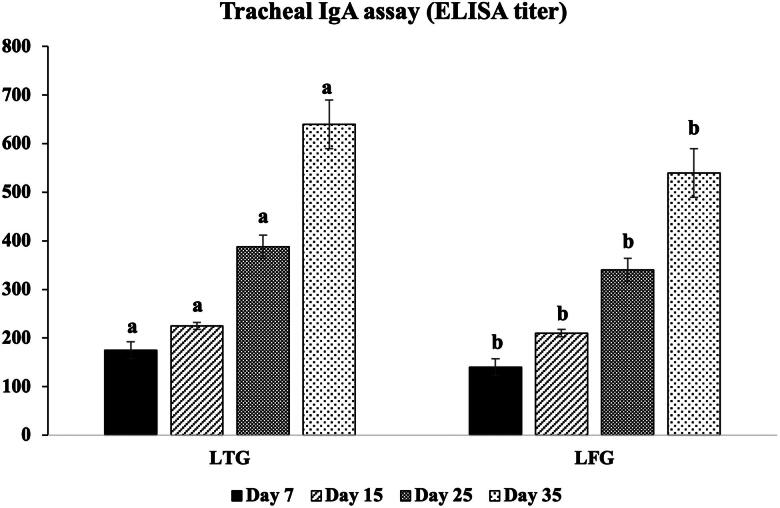
Local (IgA) immune modulation in both groups. Different letters above the bars indicate statistically significant difference between groups at *p* value below 0.05.

The opsonic activity (MΦ and phagocytic index) was significantly greater (*p ≤* 0.001) in LTG compared to LFG, with MΦ counts of 10^5^,10^6^ vs.10^3^, 10^4^ and phagocytic index as 6.8, 8.2 vs. 3.5, 4.8 at 25 and 35 days, respectively (Fig. 2a, b). Significantly higher (*p* ≤ 0.001) local IgA was recorded (640 vs. 540 ELISA units at 35 days), indicating a high immune modulation in LTG in relation to LFG (Fig. 3).

### Humoral immunity

**Figure 4.**
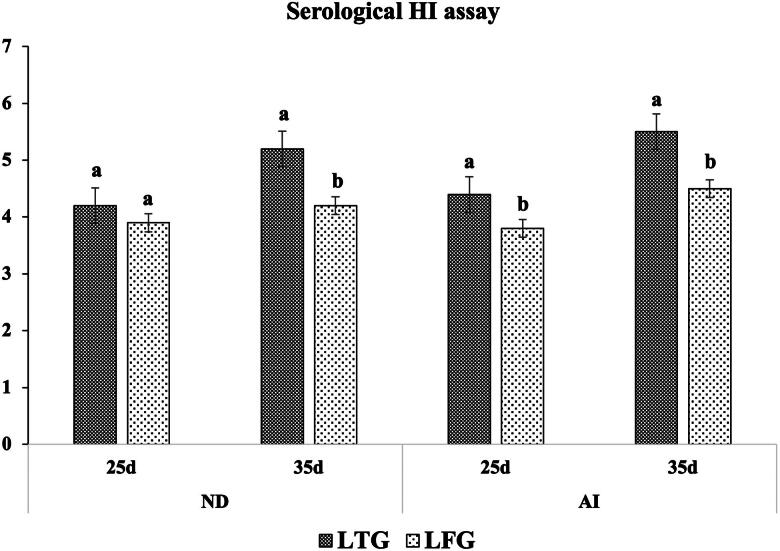
Humoral (HI titers) immune modulation in both groups. Different letters above the bars indicate statistically significant difference between groups at *p* value below 0.05.

Higher HI titers for both ND and HPAI-H5N1 were reported, indicating a significantly higher (*p* ≤ 0.01) immune modulation in LTG. At 35 days, the HI titers for ND and H5N1 reached 5.2 and 5.5 vs. 4.2 and 4.5 in LTG and LFG, respectively (Fig. 4).

## Discussion

Lysozyme has a significant antibacterial effect on both Gram negative and Gram positive bacteria by impairing DNA and RNA synthesis, activating autolysin production, permeabilizing cell walls and membranes, causing their depolarization, and ultimately cytosol leakage.^36^^,^^37^ By altering the intestinal histology and exerting an influence on the enzyme expression and metabolism of the cecal microbiota, the use of lysozyme as a feed supplement enhanced the growth performance of broiler chickens.^38^ According to an *in-vitro* study by Zhang et al.^39^ 200 μg/ml of lysozyme prevented *Clostridium perfringens* from growing as well as the synthesis of the toxin that results in the lesions linked to necrotic enteritis (NE) in chickens. According to certain *in-vivo* investigations, lysozyme could be a good option to get rid of *Clostridium perfringens* and boost broiler chicken development.^40^ However, extended *in vivo* research are urgently required to determine the best doses of lysozyme to use in poultry instead of antibiotics and to identify the critical times of the broiler growth cycle when lysozyme may have the biggest effects on growth performance and the microbiota populations of the gastrointestinal tract (GIT) of broiler chickens.^41^ Generally, the supplementation with lysozyme could support poultry industry with several benefits such as providing antibiotic free poultry products, enhancing innate and adaptive immunity, gut integrity, alongside with anti-inflammatory and antioxidant effects in broiler chickens.^42^

In the same direction, the use of a microbial lysozyme (Lysonir®) in commercial broiler chickens through both drinking water (thrice) and spray (once), novel methods of application was evaluated in this study. Our results obviously indicated significant high (*p* ≤ 0.05) performance parameters regarding FCR,and EPEF which could be related to better intestinal integrity score and significant higher (*p* ≤ 0.05) intestinal *Lactobacillus* counts in lysozyme treated group (LTG) compared to lysozyme free group (LFG). Similar results were obtained by Abdel-Latif et al.^19^ who reported significant (*p* < 0.05) improvements with the dietary supplementation of exogenous lysozyme (with a dose of 90 g/ton) for broiler chickens such as 5.39% in a growth rate or AFBWG (without difference in feed intake), 6.1% in FCR, and 17.2% in EPEF. The same results were supported by Gong et al.^43^ who determined the effect of 100 ppm LYZ as feed additive on growth performance and intestinal microbiota of broiler chickens in each period of the growth cycle using new or used litter and concluded that LYZ supplementation had changed the intestinal microbiota of broiler chickens through reducing the number of harmful bacteria.

*Lactobacillus* and *Bifidobacterium* are the two key helpful genus of bacteria in the GIT of birds (Fooks and Gibson, 2002), while the main pathogenic gut bacteria of poultry are *Clostridium*, *Escherichia coli*, *Salmonella*, and *Camplylobacter*.^44^ Abdel-Latif et al.^19^ and Gong^42^ recorded an enhancement in the gut microbiota through significant decreases (*p* < 0.01) in the damaging fecal *Coliform* and *Clostridia* and an increase (*p* < 0.05) in the valuable *Lactobacillus* counts. Similar results were indicated not only in chickens but also in rabbits by El-Deep et al.^45^ who reported that LYZ (200 mg per kg diet) enhanced the growth performance (*p* < 0.05), reduced feed intake and FCR, improved the hematological and serum biochemical parameters, linearly reduced (*p <* 0.05) the total count of Escherichia coli and Clostridium was decreased and considerably increased the Lactobacilli count in rabbits.

The maintenance of a healthy microbiota within the GIT environment has a strong relationship with the bird’s health, well-being, and productivity.^46^ This might be the actual situation of the broiler chickens in LTG in our study as the hydrolytic enzyme, lysozyme, could protect the birds against these harmful bacteria through its hydrolysis compromising direct action of the integrity of the bacterial cell wall, causing its lysis.^16^^,^^18^ Liu et al.^41^ also mentioned that exogenous lysozyme declined *C. perfringens* colonization and increased the intestinal barrier function and growth performance of chickens. In another study by Xia et al.^47^, the lysozyme supplementation led to a significant (*p* < 0.05) enrichment of genes involved in the synthesis/degradation of bacterial outer membranes and cell walls, cross-cell substrate transport, and carbohydrate metabolic processes, promoting the cecal microbiota carbon and energy metabolism. However, this did not contribute significantly (*p* > 0.05) to the growth, and different compositions of the bacterial and fungal communities of cecal microbiota in broiler chickens. In this study, the improvement in intestinal integrity score between LTG vs. LFG was a direct result of lysozyme treatment (*p* ≤ 0.05) as recorded previously by Du and Guo^48^ who proved that lysozyme or essential oils decreased the mortality, improved the intestinal integrity, alleviated the gut lesions, and significantly reduced the ileal concentration of sialic acid and the Mucin2 mRNA expression following *C. perfringens* infection in chickens.

Additionally, according to several studies,^17^^,^^42^^,^^49^^,^^50^ lysozymes are linked to indirect bacteriolytic activity through stimulating macrophage phagocytic function. The immunoglobulins IgG, IgM, and IgA found in poultry peripheral blood at the greatest levels are significant indicators of the humoral immune system’s functionality.^51^ Herein, greater immunomodulations were demonstrated in LTG compared to LFG by raising cellular (opsonic activity through MΦ and phagocytic index), local (IgA) and humoral (HI titers for ND and HPAI-H5N1) immune responses. Abdel-Latif et al.^19^ indicated that the gut nonspecific immunity biomarkers expression (*INF-*γ, *IL-10*, and *IL-18*), and serum globulin levels were significantly elevated in lysozyme-supplemented groups (especially LYZ90; 90 mg lysozyme/Kg diet) which confirmed the enhancement of the innate (nonspecific) immunity that was reflected on the increase of HI titers for ND. Several research evidences showed that lysozyme plays an important role not only in defense mechanism but also in regulation and/or activation of immune response mitigating the inflammatory response.^14^^,^^27^^,^^52–57^ The enhancement of macrophage-dependent innate immunity through the activation of specific cathelicidins eliciting a pronounced immune response.^56^,^58^

Lysozyme has anti-inflammatory properties in addition to its antibacterial activity through a gene-regulatory system involving inflammatory pathway proteins such IL-1β and tumor necrosis factor alpha (TNF-α). Ibrahim et al.^24^ recorded, for the first time, that lysozyme peptides could antagonize the pathogen-induced inflammatory response through the significant reduction in pro-inflammatory cytokines such as IL-1β, TNF-α and IL-6. Also, the *in-vitro* supplementation of lysozyme in a monocyte cell line, a kind of cell that interacts with lysozyme, supported the evidence the anti-inflammatory action of lysozyme on the basis of transcriptomic regulation data resulting from the broad perspective of a whole-transcriptome profiling.^25^ Lysozyme, used orally, induced an anti-inflammatory effect *in-vitro* and *in-vivo* through mitigating the phosphorylation of JNK, and significant reduction in the amounts of IL-6 and TNF-α in sera.^26^ The obtained anti-inflammatory effect appeared without inhibiting innate immune responses of macrophages. Not only in animals, but also in human, the use of lysozyme was effective in the treatment of inflammation through minimizing the pro-inflammatory mediators pathways such as IL-1β, IL-6 TNF-α and induction of immunomodulation.^25^^,^^26^^,^^59–61^ Herein, a considerable reduction in pro-inflammatory cytokine levels of IL-1β and CXCL8 throughout the experimental period suggested that the inflammatory status of the birds had been minimized. This is another way by which the lysozyme (Lysonir®) treated birds performed better.

## Conclusion

Exogenous aqueous microbial lysozyme (Lysonir^®^) prophylactic protocol in drinking water and spray, as innovative methods, had multiple beneficial effects in commercial broiler chickens such as postbiotic enhancement of feed efficiency, intestinal integrity, and intestinal *Lactobacillus* counts as well as improvement of the anti-inflammatory, local, cellular, and humoral immune, responses.

## Data Availability

The data of this study are available from the corresponding authors, [MAA & AO], upon reasonable request.
